# Frailty Phenotype Among Older Adults Receiving SGLT-2 Inhibitors in a CKD-Predominant Population: A Prospective Cross-Sectional Study

**DOI:** 10.3390/medicina62101938

**Published:** 2026-10-08

**Authors:** Mehmet Sezen, Onder Bugra Kaynarca

**Affiliations:** 1 Nephrology Clinic, Bursa City Hospital, 16110 Bursa, Türkiye; 2 Department of Nephrology, Faculty of Medicine, Yalova University, 77200 Yalova, Türkiye; 3 Department of Internal Medicine, Bursa City Hospital, 16110 Bursa, Türkiye; onderbugrak@gmail.com

**Keywords:** frailty, pre-frailty, older adults, chronic kidney disease, SGLT-2 inhibitors

## Abstract

*Background and Objectives:* Frailty is a multisystem clinical syndrome characterized by reduced physiological reserve and increased vulnerability to adverse health outcomes. It is particularly relevant in older adults with chronic conditions such as chronic kidney disease (CKD). This study aimed to determine the prevalence of frailty and pre-frailty among older adults receiving sodium–glucose cotransporter-2 (SGLT-2) inhibitor therapy in a CKD-predominant population and to evaluate clinical, anthropometric, and laboratory factors associated with frailty status. *Materials and Methods:* This prospective, cross-sectional study included patients aged ≥60 years receiving SGLT-2 inhibitor therapy for at least one month who underwent frailty assessment during a routine outpatient visit. Frailty status was assessed using a prespecified, modified Fried Physical Frailty Phenotype. Anthropometric and physical performance measurements were obtained using a standardized protocol by the same nephrologist. *Results:* A total of 163 adults aged ≥60 years receiving SGLT-2 inhibitor therapy were included in this CKD-predominant cohort. Frailty and pre-frailty were identified in 26.4% (95% CI 20.2–33.6%) and 46.0% (95% CI 38.5–53.7%) of participants, respectively. In multivariable logistic regression analysis, higher BMI (aOR 1.12, 95% CI 1.03–1.22; *p* = 0.010) and higher CCI scores (aOR 1.42, 95% CI 1.12–1.81; *p* = 0.004) were independently associated with higher odds of non-robust status, whereas higher hemoglobin levels were associated with lower odds (aOR 0.72, 95% CI 0.57–0.91; *p* = 0.007). *Conclusions:* Frailty and pre-frailty were common among older adults receiving SGLT-2 inhibitor therapy in this CKD-predominant population. Higher BMI and greater comorbidity burden were independently associated with higher odds of non-robust status, whereas higher hemoglobin levels were associated with lower odds. Frailty assessment may provide complementary information regarding physical reserve and vulnerability in this population rather than serving as a tool for determining eligibility for SGLT-2 inhibitor therapy. Given the study design and absence of a comparator group, no treatment-specific conclusions regarding SGLT-2 inhibitor therapy and frailty can be drawn.

## 1. Introduction

The global population is aging rapidly, and it is estimated that one in six people worldwide will be aged 60 years or older by 2030, with the global population aged 60 years and older projected to reach 2.1 billion by 2050 [1]. Advanced age is associated with an increasing burden of chronic diseases and frailty. Although frailty is more common among older adults, it may also occur at younger ages, particularly in the presence of comorbid conditions. Chronic diseases such as chronic kidney disease (CKD), diabetes mellitus (DM), and cardiovascular disease (CVD) are commonly associated with frailty. Frailty is common among patients with CKD and becomes increasingly prevalent with declining kidney function [2,3]. In patients with CKD, frailty is associated with multiple clinical and pathophysiological factors, including chronic inflammation, protein–energy wasting, anemia, reduced physical performance, and sarcopenia [4,5,6]. CKD and frailty are therefore closely interrelated clinical conditions that frequently coexist and are associated with adverse outcomes [3,5,6,7]. Accordingly, early recognition of frailty and identification of associated factors are important clinical considerations in older adults with CKD. Frailty is a multidimensional clinical syndrome characterized by reduced physiological reserve and increased vulnerability to stressors [8,9,10]. Frailty exists along a continuum from robustness to pre-frailty and frailty and is associated with adverse outcomes, including functional decline, falls, hospitalization, and mortality [10,11,12]. Among the available assessment tools, the Fried Frailty Phenotype is one of the most widely used and defines physical frailty based on five components: unintentional weight loss, exhaustion, weakness assessed by handgrip strength, slow walking speed, and low physical activity [11]. Sodium–glucose cotransporter-2 (SGLT-2) inhibitors, initially developed as glucose-lowering agents, have evolved into a cornerstone of contemporary cardiorenal therapy. Their clinical role now extends well beyond glycemic control, with robust evidence demonstrating kidney protection and slower CKD progression in patients with CKD with and without diabetes [13,14,15]. These benefits complement established kidney-protective strategies, including renin–angiotensin system inhibition, and have substantially expanded the use of SGLT-2 inhibitors in routine nephrology practice. Consequently, older adults receiving SGLT-2 inhibitor therapy represent a contemporary and clinically relevant population with a substantial burden of kidney disease and multimorbidity, in whom physical vulnerability may coexist with cardiorenal disease and its treatment. In the present study, SGLT-2 inhibitor use therefore served to define this clinical population of interest rather than representing an exposure hypothesized to cause or prevent frailty. In this clinical context, assessment of physical frailty may provide complementary information regarding physiological reserve and vulnerability in older adults receiving SGLT-2 inhibitor therapy. Frailty has been examined in analyses of major SGLT-2 inhibitor trials [16,17,18]; however, these studies primarily evaluated treatment efficacy and safety according to frailty status rather than characterizing the Physical Frailty Phenotype and its individual components in routine clinical practice. Data describing the prevalence and component-level distribution of physical frailty among older adults receiving SGLT-2 inhibitor therapy in real-world clinical settings therefore remain limited. Accordingly, the present study aimed to determine the prevalence of frailty and pre-frailty, characterize the distribution of individual frailty components, and evaluate clinical, anthropometric, and laboratory factors associated with non-robust status among adults aged ≥ 60 years receiving SGLT-2 inhibitor therapy in a CKD-predominant outpatient population.

## 2. Materials and Methods

### 2.1. Study Design and Patients

**Figure 1:**
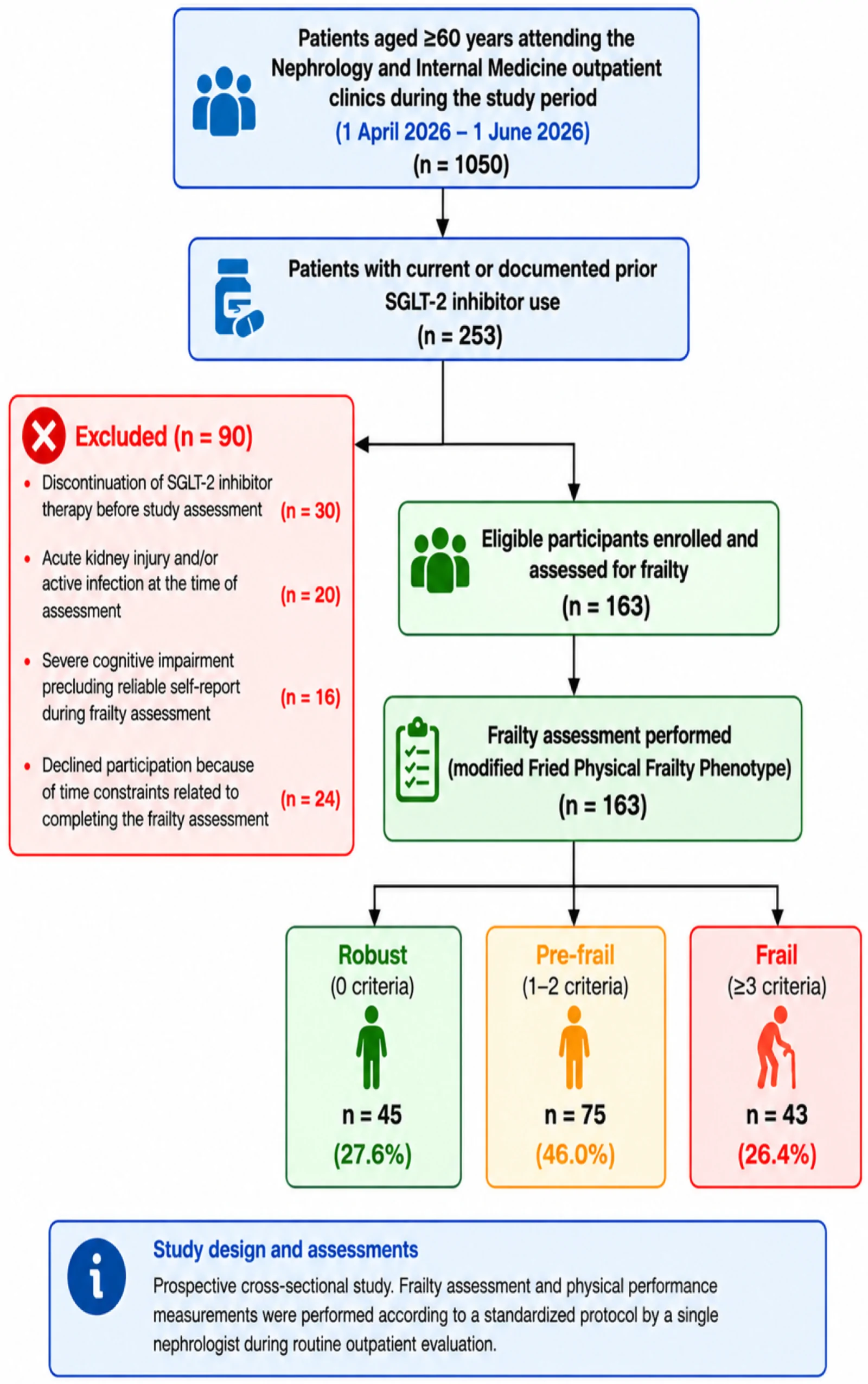
Patient selection and classification according to frailty status. Of 253 individuals with current or previous use of an SGLT-2 inhibitor who were further assessed for eligibility, 90 were excluded and 163 participants actively receiving SGLT-2 inhibitor therapy underwent frailty assessment using the modified Fried Physical Frailty Phenotype. Participants were classified as robust (*n* = 45), pre-frail (*n* = 75), or frail (*n* = 43). SGLT-2: sodium–glucose cotransporter-2.

This prospective, cross-sectional study was conducted between 1 April and 1 June 2026, at the Nephrology and Internal Medicine Outpatient Clinics of Bursa City Hospital. During the study period, 1050 adults aged ≥ 60 years attending these outpatient clinics were screened for eligibility. Among them, 253 patients reported current or previous use of an SGLT-2 inhibitor and underwent further eligibility assessment. Current SGLT-2 inhibitor use and treatment duration were ascertained by direct patient report during the study visit. Participants reporting current SGLT-2 inhibitor use were asked how long they had been receiving the treatment. Of the 253 patients, 30 who reported previous SGLT-2 inhibitor use were no longer actively receiving treatment at the time of study assessment and were therefore excluded; specific reasons for treatment discontinuation were not systematically recorded. An additional 60 patients were excluded for the following reasons: acute kidney injury and/or active infection at the time of assessment (*n* = 20), severe cognitive impairment precluding reliable self-report during frailty assessment (*n* = 16), or declining participation due to time constraints associated with completing the frailty assessment (*n* = 24). The remaining 163 eligible participants, all of whom reported that they had been actively receiving an SGLT-2 inhibitor (empagliflozin or dapagliflozin) for at least one month, were enrolled and underwent a standardized frailty assessment during the study visit. The ≥1-month treatment duration was selected as a pragmatic eligibility criterion to exclude patients who had only recently initiated SGLT-2 inhibitor therapy; this threshold was not intended to represent a minimum duration required for an effect of SGLT-2 inhibitor therapy on frailty. Of these, 138 (84.7%) were recruited from the nephrology outpatient clinic and 25 (15.3%) from the internal medicine outpatient clinic. The inclusion and exclusion criteria are summarized in Table 1, and the patient selection process is presented in Figure 1. Participants were classified as robust, pre-frail, or frail according to the modified Fried Physical Frailty Phenotype, as described below.

### 2.2. Clinical and Anthropometric Evaluation

Demographic and clinical characteristics including age, sex, smoking status, comorbidities, GFR category, type and duration of SGLT-2 inhibitor therapy, and the number of regularly used medications were prospectively recorded at the study visit. Burden of comorbidity was assessed using the Charlson Comorbidity Index (CCI). Concurrent medications, including renin–angiotensin system (RAS) inhibitors, diuretics, and finerenone, were also recorded.

Anthropometric and physical measurements, including body weight, height, body mass index (BMI), systolic and diastolic blood pressure (SBP and DBP), walking speed, and handgrip strength, were obtained during the study visit by the same nephrologist using a standardized protocol to minimize inter-observer variability. BMI was calculated as body weight in kilograms divided by the square of height in meters (kg/m^2^). Walking speed and handgrip strength were measured as part of the frailty assessment, as described below.

### 2.3. Laboratory Tests

Laboratory data obtained as part of routine outpatient evaluation were prospectively recorded. Laboratory variables included serum creatinine, estimated glomerular filtration rate (eGFR), spot urine protein-to-creatinine ratio (PCR), spot urine albumin-to-creatinine ratio (ACR), hemoglobin, serum albumin, C-reactive protein (CRP), and the neutrophil-to-lymphocyte ratio (NLR). eGFR was calculated using the 2021 Chronic Kidney Disease Epidemiology Collaboration (CKD-EPI) creatinine equation [19] and was used to classify participants according to GFR category. The study-visit eGFR alone was not used to establish CKD chronicity. CKD was defined by an eGFR < 60 mL/min/1.73 m^2^ and/or evidence of kidney damage, including persistent albuminuria (ACR ≥ 30 mg/g), present for at least 3 months. CKD chronicity was ascertained by reviewing longitudinal medical records and previous laboratory results available in the hospital electronic health record and, when these were unavailable locally, in the national electronic health record system. Previous kidney function and albuminuria measurements were reviewed to establish persistence for at least 3 months, and available renal ultrasonography findings were also reviewed for evidence of chronic structural kidney abnormalities. Accordingly, participants with G1–G2 kidney function were classified as having CKD only when persistent albuminuria or other evidence of kidney damage was documented. CKD status was classified as CKD, non-CKD, or indeterminate when available data were insufficient to establish or exclude CKD. All laboratory measurements were obtained on the same day as the frailty assessment.

### 2.4. Frailty Assessment

Frailty was prospectively assessed using the modified Fried Physical Frailty Phenotype. All frailty assessments and physical performance measurements were performed by the same nephrologist according to a standardized protocol. Assessments were based on direct participant interviews and performance-based measurements; proxy-reported information was not used.

The Fried Physical Frailty Phenotype, first described by Fried et al. [11], consists of five components: unintentional weight loss, self-reported exhaustion, low physical activity, slow gait speed, and weakness assessed by handgrip strength. Participants who met none of these criteria were classified as robust, those meeting one or two criteria as pre-frail, and those meeting three or more criteria as frail.

#### 2.4.1. Assessment of Handgrip Strength

Handgrip strength was measured using a hydraulic hand dynamometer (Saehan SH5001, Saehan Corporation, Changwon, Republic of Korea). The dominant hand was identified before testing, and three consecutive measurements were obtained from the dominant hand for each participant. The highest value was used for the frailty assessment. Weakness was defined according to the sex- and BMI-specific cut-off values of the original Fried frailty phenotype [11]. For men, weakness was defined as handgrip strength ≤ 29 kg for BMI ≤ 24 kg/m^2^, ≤30 kg for BMI 24.1–26.0 kg/m^2^, ≤30 kg for BMI 26.1–28.0 kg/m^2^, and ≤32 kg for BMI > 28.0 kg/m^2^. For women, the corresponding cut-off values were ≤17 kg for BMI ≤ 23 kg/m^2^, ≤17.3 kg for BMI 23.1–26.0 kg/m^2^, ≤18 kg for BMI 26.1–29.0 kg/m^2^, and ≤21 kg for BMI > 29.0 kg/m^2^.

#### 2.4.2. Assessment of Gait Speed

Gait speed was assessed using a 5 m walking test performed at the participant’s usual walking pace. Slow walking was defined using the sex- and height-specific thresholds of the original Fried Frailty Phenotype [11]. Because the original Fried criteria were based on a 15 ft (4.57 m) walking test, the corresponding gait-speed thresholds were calculated and applied to the 5 m walking test. The original thresholds of ≥7 s and ≥6 s over 15 ft corresponded to gait speeds of ≤0.65 m/s and ≤0.76 m/s, respectively. Accordingly, slow walking was defined as a gait speed of ≤0.65 m/s for men ≤ 173 cm in height and women ≤ 159 cm in height, and ≤0.76 m/s for men > 173 cm in height and women > 159 cm in height.

#### 2.4.3. Assessment of Unintentional Weight Loss

Unintentional weight loss was assessed by self-report and was defined as an unintentional loss of >4.5 kg (10 lb) or ≥5% of body weight during the previous year, according to the original Fried Frailty Phenotype [11].

#### 2.4.4. Assessment of Exhaustion

Exhaustion was assessed according to the original Fried Frailty Phenotype using two self-reported items derived from the Center for Epidemiologic Studies Depression (CES-D) scale. Participants were asked whether they felt that “everything I did was an effort” or that “I could not get going” during the previous week. Participants who scored 2 (moderate amount of time; 3–4 days) or 3 (most of the time; 5–7 days) on either of the two items were considered to meet the exhaustion criterion [11].

#### 2.4.5. Assessment of Physical Activity

Low physical activity was assessed using a prespecified, study-specific self-reported approach rather than the original energy expenditure-based physical activity criterion of the Fried phenotype. The three items were selected a priori to capture complementary aspects of habitual physical activity that could be feasibly assessed in the outpatient setting, including regular walking, engagement in daily activities, and sedentary behavior, thereby providing a pragmatic assessment of both active and sedentary aspects of habitual daily activity. The selection of these domains was informed by previous modified Fried phenotype approaches in which the physical activity component was operationalized using simplified measures of habitual physical activity [20,21]. However, the specific three-item combination and the ≥2/3 threshold used in the present study were prespecified and study-specific and have not been validated against the original energy expenditure-based Fried criterion. Participants were asked three questions: (1) whether they had walked for at least 20–30 min on ≥5 days during the preceding week; (2) whether they were generally active in daily activities such as housework, shopping, and going outdoors; and (3) whether they spent most of the day sitting or lying down. Indicators of low physical activity were defined as not meeting the specified walking frequency, lack of activity in daily life, and predominantly sedentary behavior, respectively. Participants meeting at least two of these three indicators were classified as having low physical activity, as prespecified in the study protocol. The exact wording of the three questions and the scoring algorithm are provided in Supplementary Table S1.

### 2.5. Ethics Committee Approval

The study was conducted in accordance with the Declaration of Helsinki and approved by the Bursa City Hospital Ethics Committee (protocol code 2026-03/15; date of approval: 4 February 2026). Written informed consent was obtained from each participant before the initiation of study procedures. The study was not prospectively registered in ClinicalTrials.gov or another study registry because it was designed as a non-interventional, observational cross-sectional study without assignment of participants to an intervention.

### 2.6. Statistical Analysis

Sample size was calculated using the formula for estimating a single proportion. Based on a frailty prevalence of 27% reported in outpatients with advanced CKD assessed with the Fried phenotype [22], a minimum of 155 participants was required to estimate prevalence with ±7% precision at a 95% confidence level; the final sample of 163 met this requirement. Prevalence estimates are reported with Wilson 95% confidence intervals.

The distribution of continuous variables was assessed for normality using the Shapiro–Wilk test. Continuous variables were presented as mean ± standard deviation or median (Q1–Q3), whereas categorical variables were presented as *n* (%). Comparison between two groups were performed using the independent-samples *t*-test for normally distributed variables; otherwise, the Mann–Whitney U test was used. For comparisons involving more than two groups, one-way analysis of variance (ANOVA) was used for normally distributed variables, whereas the Kruskal–Wallis test was used for non-normally distributed variables. When the overall ANOVA or Kruskal–Wallis test was significant, post hoc pairwise comparisons were performed using the Bonferroni test after ANOVA and the Dunn–Bonferroni test after the Kruskal–Wallis test, respectively. Categorical variables were compared using Pearson’s chi-square or Fisher–Freeman–Halton tests, with Bonferroni-corrected column proportion tests for pairwise comparisons. Effect sizes were reported as η^2^, ε^2^, r, Cohen’s d, φ, or Cramér’s V, as appropriate. Analyses were performed using available data without imputation.

Factors associated with non-robust status (pre-frail and frail combined) were examined using binary logistic regression. Variables with *p* < 0.25 in univariate analysis were considered candidates (Supplementary Table S2), and the number of variables was limited according to the events-per-variable principle; age and sex were forced into the model, and CCI was used instead of individual comorbidities to avoid collinearity. Multicollinearity was assessed using variance inflation factors (VIFs). Model performance was assessed using the Hosmer–Lemeshow test, Nagelkerke R^2^ and AUC. No covariate data were missing in the multivariable model. Influential observations were examined using Cook’s distance, leverage, and studentized residuals. Sensitivity analyses were performed after excluding the participant with indeterminate CKD status and after restricting the analysis to participants meeting CKD criteria, and ordinal logistic regression was used with the Fried score (0–5) as the outcome.

Analyses were performed using IBM SPSS Statistics, Version 25.0 (IBM Corp., Armonk, NY, USA), and *p* < 0.05 was considered statistically significant.

### 2.7. Use of Generative Artificial Intelligence

Generative artificial intelligence (ChatGPT, OpenAI, GPT-5.6 Sol) was used solely to assist with the visual structuring, data layout, and diagram design of the study flowchart (Figure 1) and the graphical abstract. The scientific content, numerical data, statistical analyses, and interpretation of the study findings were determined and verified by the authors. The authors reviewed and edited all AI-assisted outputs and take full responsibility for their accuracy and content.

## 3. Results

A total of 163 adults aged ≥60 years receiving an SGLT-2 inhibitor were enrolled. The median age was 69 years (Q1: 65 years; Q3: 73 years), 52.1% were male, and the mean BMI was 31.44 ± 5.52 kg/m^2^. The mean number of medications used by the participants was 7.83 ± 2.47. Diabetes mellitus and hypertension were the most common comorbid conditions (*n* = 143, 87.7% for both), and the median CCI was 3. CKD status could be determined in 162 of 163 participants: 137 (84.6%) met the criteria for CKD and 25 (15.4%) were classified as non-CKD. CKD status remained indeterminate in one participant because the available longitudinal data were insufficient to establish or exclude CKD. Regarding eGFR categories, 67 participants (41.1%) were classified as G3b. The most commonly used SGLT-2 inhibitor was empagliflozin (55.8%), followed by dapagliflozin (44.2%), and 58.3% of participants had been receiving SGLT-2 inhibitor therapy for >12 months. The demographic, clinical, and laboratory characteristics of the overall study population are summarized in Table 2. Missing data were minimal and limited to urine ACR (*n* = 4), CRP (*n* = 3), urine PCR (*n* = 1), serum albumin (*n* = 1), and CKD status (*n* = 1); analyses were performed on available data (Supplementary Table S3).

According to the modified Fried Physical Frailty Phenotype, 45 (27.6%; 95% CI, 21.3–34.9%) participants were classified as robust, 75 (46.0%; 95% CI, 38.5–53.7%) as pre-frail, and 43 (26.4%; 95% CI, 20.2–33.6%) as frail. The groups were significantly different from each other in terms of age distribution, BMI, SBP and CCI. Patients in the frail group were significantly older and had higher CCI values (*p* < 0.001 for both); however, SBP was highest in the pre-frail group (*p* = 0.043). Mean BMI also differed significantly between the groups (*p* = 0.024). Post hoc analysis with Bonferroni correction showed that pre-frail participants had a significantly higher BMI than robust participants (32.51 ± 5.22 vs. 29.68 ± 4.75 kg/m^2^, *p* = 0.019), whereas no significant difference was observed between frail and robust (*p* = 0.416) or frail and pre-frail participants (*p* = 0.856). The proportions of patients with HF and COPD were significantly higher in the frail group (*p* = 0.017 and *p* = 0.034, respectively). With regard to the laboratory parameters, a significant difference was observed between the groups in terms of median PCR, ACR, NLR, hemoglobin and serum albumin values (all *p* < 0.05). Median PCR and ACR values were highest in frail participants, whereas pre-frail participants had the lowest values. Hemoglobin and serum albumin concentrations were lowest, whereas NLR values were highest, in frail participants. Serum creatinine, CRP and eGFR values were not significantly different between the groups. The distribution of albuminuria categories differed significantly between the groups (*p* = 0.011). Post hoc column proportion comparisons with Bonferroni correction showed that the proportion of participants with normoalbuminuria (A1) was significantly lower in the frail group (21.4%) than in both the robust (50.0%) and pre-frail (52.1%) groups, whereas moderately increased albuminuria (A2) was more frequent in frail than in pre-frail participants (50.0% vs. 23.3%; *p* < 0.05). The proportion of participants with severely increased albuminuria (A3) did not differ between the groups (*p* > 0.05). Frailty prevalence according to eGFR category was 20.5% in G1–G2, 12.5% in G3a, 29.9% in G3b and 50.0% in G4–G5 (*p* = 0.097; *p* for trend = 0.056). Detailed comparisons of demographic, clinical, and laboratory characteristics according to frailty status are presented in Table 3.

**Figure 2:**
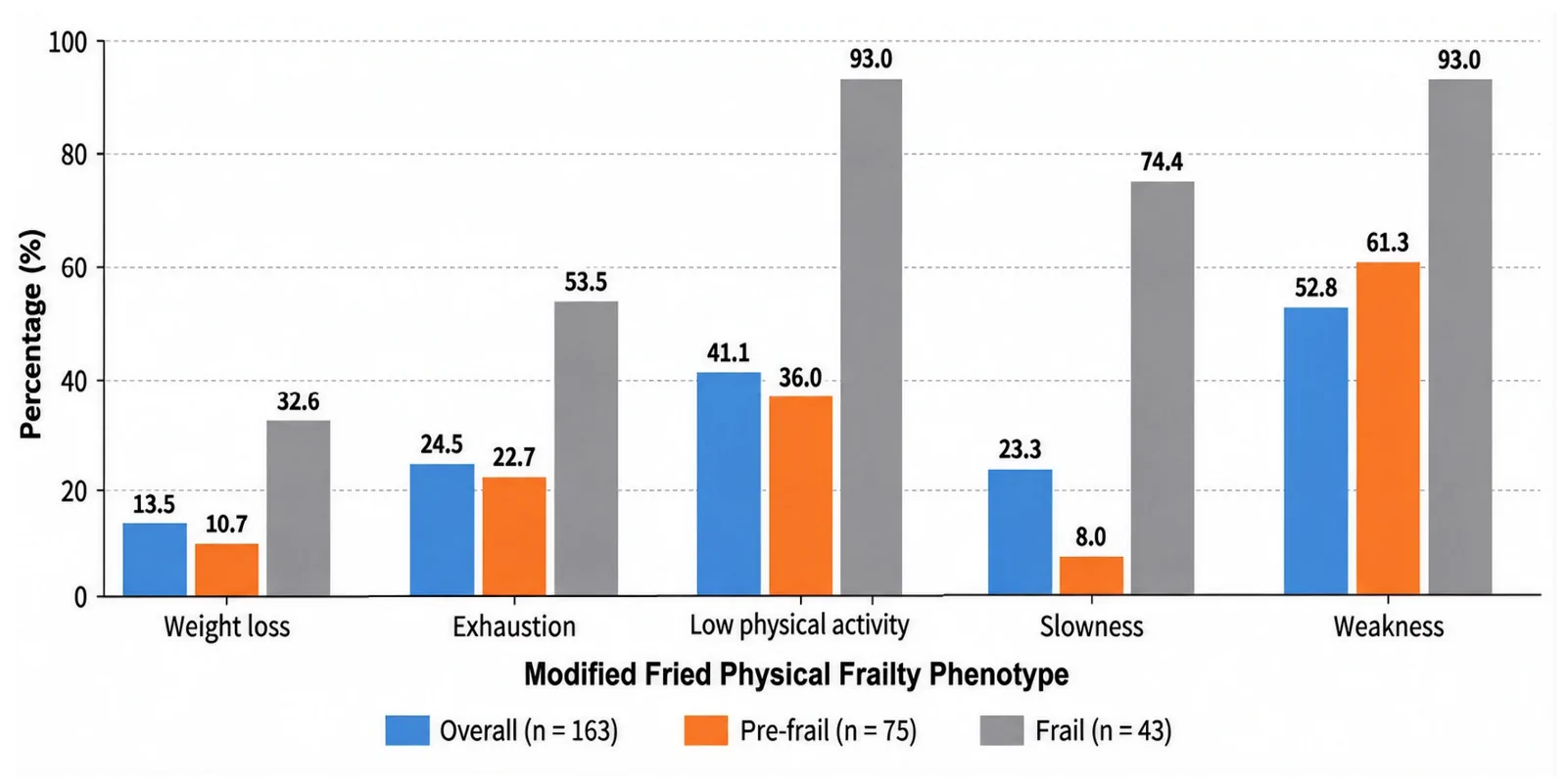
Distribution of individual components of the modified Fried Physical Frailty Phenotype according to frailty status (*n* = 163). Participants were classified as robust (*n* = 45), pre-frail (*n* = 75), or frail (*n* = 43). The low physical activity component was assessed using the study-specific three-item self-reported approach described in Section 2. Values are presented as percentages.

Among pre-frail participants, weakness was the most prevalent component (61.3%) of the modified Fried Physical Frailty Phenotype, followed by low physical activity (36.0%). Among frail participants, weakness and low physical activity were equally the most prevalent components (93.0% each). In the overall study cohort, weakness was also the most prevalent component (52.8%), followed by low physical activity (41.1%) and exhaustion (24.5%) (Figure 2).

The distribution of weakness according to the sex- and BMI-specific strata of the original Fried Frailty Phenotype is presented in Supplementary Table S4. Among men, weakness was present in 6/10 (60.0%), 1/4 (25.0%), 5/19 (26.3%), and 31/52 (59.6%) participants with BMI ≤ 24, 24.1–26.0, 26.1–28.0, and >28.0 kg/m^2^, respectively. Among women, no participants had a BMI ≤23 kg/m^2^; weakness was present in 4/6 (66.7%), 5/15 (33.3%), and 34/57 (59.6%) participants with BMI 23.1–26.0, 26.1–29.0, and >29.0 kg/m^2^, respectively. In sensitivity analyses, 28 of the 75 pre-frail participants (37.3%; 23.7% of all non-robust participants) met only the weakness criterion. When the physical activity component was excluded, 53 (32.5%) participants were classified as robust, 92 (56.4%) as pre-frail, and 18 (11.0%) as frail. Using Turkish grip-strength cut-offs, 62 (38.0%) participants were classified as robust, 71 (43.6%) as pre-frail, and 30 (18.4%) as frail (Supplementary Table S5). Weakness did not differ significantly across BMI categories (*p* = 0.134), and weight loss did not differ significantly across SGLT-2 inhibitor therapy duration categories (*p* = 0.40).

Participants were further categorized as robust (*n* = 45) and non-robust (pre-frail + frail; *n* = 118). Compared with robust participants, non-robust participants had significantly higher CCI scores (median 3 vs. 2, *p* = 0.001), lower hemoglobin levels (12.96 ± 1.99 vs. 14.23 ± 1.74 g/dL, *p* < 0.001), and lower serum albumin concentrations (median 43.9 vs. 45.3 g/L, *p* = 0.001). No significant differences were observed between the two groups regarding age, heart failure, COPD, PCR, ACR, NLR, eGFR, GFR category, CKD status, albuminuria category, SGLT-2 inhibitor type, or duration of SGLT-2 inhibitor therapy (Table 4).

In univariate analysis, sex, BMI, hypertension, diabetes mellitus, heart failure, COPD, CCI, number of medications, hemoglobin, serum albumin, log-ACR, and log-PCR met the screening criterion of *p* < 0.25, whereas age, eGFR and NLR did not (Supplementary Table S2). In the multivariable model including age, sex, BMI, CCI and hemoglobin, higher BMI (aOR 1.12, 95% CI 1.03–1.22, *p* = 0.010) and higher CCI (aOR 1.42, 95% CI 1.12–1.81, *p* = 0.004) were independently associated with non-robust status, whereas each 1 g/dL increase in hemoglobin was associated with 28% lower odds of non-robust status (aOR 0.72, 95% CI 0.57–0.91, *p* = 0.007). Age and sex were not independently associated with non-robust status (Table 5). The Hosmer–Lemeshow test showed no evidence of poor model fit (*p* = 0.695), with a Nagelkerke R^2^ of 0.244 and an AUC of 0.761 (95% CI 0.681–0.842). Variance inflation factors ranged from 1.08 to 1.51. In a sensitivity analysis additionally adjusting for log-ACR (*n* = 159), BMI, CCI, and hemoglobin remained independently associated with non-robust status, whereas log-ACR was not (aOR 1.07, 95% CI 0.86–1.33, *p* = 0.534) (Supplementary Table S6). The maximum Cook’s distance was 0.05, and exclusion of observations with Cook’s distance >4/n did not materially change the conclusions (Supplementary Table S7). The results were also similar after excluding the participant with indeterminate CKD status (*n* = 162) or restricting the analysis to participants meeting CKD criteria (*n* = 137). In ordinal logistic regression using the Fried score, higher CCI (OR 1.41, 95% CI 1.20–1.66) and lower hemoglobin (OR 0.78, 95% CI 0.65–0.94) remained associated with higher frailty scores, whereas the association with BMI was attenuated (OR 1.04, 95% CI 0.98–1.10) (Supplementary Table S7).

## 4. Discussion

In this cross-sectional study of adults aged ≥60 years receiving SGLT-2 inhibitors in a CKD-predominant population, we evaluated the prevalence of frailty and pre-frailty using the modified Fried Physical Frailty Phenotype and investigated factors associated with non-robust status. Frailty and pre-frailty were common in this cohort. Only 27.6% of participants were classified as robust, whereas 72.4% were classified as non-robust. Higher BMI and CCI scores were independently associated with higher odds of non-robust status, whereas higher hemoglobin levels were associated with lower odds of non-robust status. Among the individual components of the modified Fried Physical Frailty Phenotype, weakness and low physical activity were the most prevalent, particularly among pre-frail and frail participants. Notably, weakness was the most frequent component among pre-frail participants (61.3%).

### 4.1. Community Prevalence

Frailty has become an increasingly important public health concern with the aging of the global population and is recognized as a major geriatric syndrome associated with adverse clinical outcomes. In the present study, frailty and pre-frailty were identified in 26.4% and 46.0% of participants, respectively.

The reported prevalence of frailty varies considerably according to the population studied and the assessment instrument used. In a systematic review of 21 community-based studies involving approximately 61,500 older adults, frailty prevalence ranged from 4.0% to 59.1%, with average prevalences of 10.7% for frailty and 41.6% for pre-frailty. Among studies using the Fried Frailty Phenotype, frailty prevalence ranged from 4.0% to 17.0%, with average prevalences of 9.9% for frailty and 44.2% for pre-frailty [23]. Similarly, a systematic review and meta-analysis of 240 population-level studies from 62 countries reported frailty and pre-frailty prevalences of 12% and 46%, respectively, among studies using physical frailty measures [24]. While the prevalence of pre-frailty was nearly identical to that observed in our cohort (46.0%), frailty was more frequent in our study (26.4% vs. 12%). This difference may reflect the greater burden of CKD and comorbidities and the clinical rather than community-based nature of our study population.

The influence of the assessment method on reported prevalence has also been demonstrated. In a systematic review of community-dwelling adults in India, frailty prevalence was lower when assessed using the Fried phenotype than when assessed using a frailty index (31% vs. 48%) [25]. A recent community-based study from Türkiye using the FRAIL Scale reported frailty and pre-frailty prevalences of 24.5% and 44.2%, respectively, among adults aged ≥65 years [26]. Despite differences in frailty assessment methods and in the characteristics and settings of the study populations, these rates were broadly comparable to those observed in our cohort (26.4% and 46.0%, respectively). This consideration is particularly relevant to the present study because the physical activity component was assessed using a study-specific three-item approach rather than the original energy expenditure-based Fried criterion, which may have influenced frailty classification and limits direct comparison with studies using the original phenotype.

### 4.2. Frailty Prevalence in Chronic Kidney Disease

In the present study, frailty and pre-frailty were identified in 26.4% and 46.0% of older adults receiving SGLT-2 inhibitor therapy in a CKD-predominant population, respectively. Previous evidence indicates a substantial burden of frailty among individuals with CKD, with prevalence generally increasing as kidney function declines [3,27,28]. Reported frailty prevalence in CKD populations is generally higher than that observed in community-dwelling older adults [3,23,28]. Across studies involving patients with CKD, reported prevalence estimates vary considerably according to population characteristics, kidney disease severity, and the frailty assessment method used [2,3,29]. In our cohort, median eGFR showed a numerical decline across frailty categories, from 49.4 mL/min/1.73 m^2^ in robust participants to 47.5 mL/min/1.73 m^2^ in pre-frail participants and 40.5 mL/min/1.73 m^2^ in frail participants; however, this difference did not reach statistical significance (*p* = 0.056).

A recent systematic review and meta-analysis including 139 studies and 1,675,482 participants reported pooled prevalences of 34.5% for frailty and 39.4% for pre-frailty among patients with CKD [30]. Similarly, in a cross-sectional study of 5888 adults aged > 65 years, frailty was more prevalent among individuals with chronic renal insufficiency than among those without it (15% vs. 6%), and this association remained significant after adjustment for comorbidity burden [29]. These findings from previous studies suggest that the relationship between impaired kidney function and frailty may not be explained solely by the burden of coexisting diseases. A substantial burden of frailty has also been reported in populations with advanced CKD and kidney failure. In a prospective cohort including patients with advanced CKD and those receiving hemodialysis, frailty and pre-frailty were identified in 36.3% and 46.5% of participants, respectively, using the Fried Frailty Phenotype [22]. Similarly, a scoping review of 164 studies comprising 116,005 participants demonstrated a high burden of frailty across nephrology populations, with prevalence estimates varying widely according to the population studied and the assessment instrument used [31]. Collectively, these findings highlight the high prevalence of frailty in advanced CKD and dialysis populations, while also demonstrating considerable heterogeneity across studies.

The prevalence observed in our cohort fell within the range of previously reported estimates in CKD populations. However, direct comparisons should be interpreted cautiously because our study included a CKD-predominant population receiving SGLT-2 inhibitor therapy rather than an exclusively CKD-defined cohort. The marked heterogeneity in the reported prevalence of frailty and pre-frailty across studies may reflect differences in study design, eligibility criteria, kidney function, demographic characteristics, comorbidity burden, and the instruments and operational definitions used to assess frailty.

### 4.3. Comorbidity Burden and Frailty

Greater comorbidity burden was independently associated with non-robust status in our cohort. Each one-point increase in the CCI was associated with 1.42-fold higher odds of non-robust status in multivariable logistic regression analysis. This association is consistent with previous evidence linking greater comorbidity burden to frailty in nephrology populations. In a multicenter cross-sectional study of patients receiving hemodialysis, Gong et al. demonstrated that higher CCI scores were independently associated with frailty in multivariable logistic regression analysis [32]. Similarly, Drost et al. reported that a CCI score ≥5 was independently associated with frailty (OR 2.6, 95% CI 1.0–6.6) in a cohort of patients with end-stage renal disease, including those receiving pre-dialysis care, hemodialysis, or peritoneal dialysis [33]. Although these studies involved patients with more advanced kidney disease than those in our CKD-predominant cohort, the association between greater comorbidity burden and frailty was consistent across different nephrology populations. Our finding should therefore be interpreted primarily as confirmatory rather than as a novel association specific to patients receiving SGLT-2 inhibitor therapy.

### 4.4. Hemoglobin and Frailty

Lower hemoglobin levels were also independently associated with non-robust status in our cohort. This finding is consistent with previous evidence linking anemia and frailty in older populations. In a large community-based study of 13,175 adults aged ≥ 50 years, Ruan et al. reported that anemia remained independently associated with frailty after adjustment for potential confounders [34]. Although that study was conducted in a community-dwelling population and used a different frailty assessment approach, its findings support an association between anemia and frailty. The observed association between lower hemoglobin levels and frailty is biologically plausible within the broader context of CKD and multimorbidity. In CKD, skeletal muscle dysfunction and physical vulnerability may reflect multiple interrelated processes, including chronic inflammation, oxidative stress, metabolic disturbances, and protein–energy wasting. Anemia may further contribute to this vulnerability through reduced oxygen delivery to skeletal muscle, potentially promoting fatigue, reduced exercise capacity, and impaired physical performance. In older adults with multiple comorbidities, the cumulative burden of these overlapping processes may reduce physiological reserve and increase susceptibility to frailty [2,35]. In a previous study of older patients with advanced CKD, frailty was independently associated with malnutrition–inflammation status, whereas overall frailty was not associated with circulating inflammatory cytokines [36].

### 4.5. Distribution of Fried Physical Frailty Phenotype Components

Among the individual components of the modified Fried Physical Frailty Phenotype, weakness was the most frequent component in the overall cohort (52.8%) and among pre-frail participants (61.3%), followed by low physical activity (41.1% and 36.0%, respectively). Among frail participants, weakness and low physical activity were the two most prevalent components, each occurring in 93.0% of participants. However, the high prevalence of weakness should be interpreted in light of the operational definition used. In the primary analysis, weakness was defined using the sex- and BMI-specific grip-strength thresholds of the original Fried phenotype. When sex-specific Turkish grip-strength cut-offs proposed by Doğan Varan et al. were applied in a sensitivity analysis, the prevalence of weakness decreased from 52.8% to 23.3%, indicating that the observed frequency of weakness was sensitive to the thresholds applied [37]. Nevertheless, impaired muscle strength and physical function are well-recognized features of frailty and sarcopenia in CKD populations [38]. The distribution and clinical relevance of individual frailty components may vary across CKD populations. In patients with advanced CKD, Nixon et al. demonstrated that exhaustion was the only Fried phenotype component significantly associated with all domains of health-related quality of life [39]. In another cohort including patients with advanced CKD and those receiving hemodialysis, Kennard et al. reported slowness as the most frequent component, followed by exhaustion, low physical activity, and weakness [22]. These differences may reflect variations in age, kidney disease severity, comorbidity burden, functional status, and the operational assessment of individual frailty components across studies. Higher BMI was independently associated with non-robust status in the multivariable analysis. This finding warrants cautious interpretation because the original Fried weakness criterion incorporates sex- and BMI-specific grip-strength thresholds, and BMI therefore contributes to the operational definition of one component of the frailty phenotype. Although the proportion of participants classified as weak did not show a consistent monotonic increase across the BMI strata used for the Fried criterion (Supplementary Table S4), some strata contained relatively few participants, limiting further interpretation. Therefore, the observed association between higher BMI and non-robust status cannot be attributed solely to the BMI-dependent weakness thresholds, although some influence of the classification approach cannot be excluded. A biological association between higher BMI and frailty is also plausible, although BMI alone does not distinguish adiposity from lean mass. Sarcopenic obesity, characterized by the coexistence of obesity and sarcopenia, has been associated with a higher likelihood of frailty in older adults [40]. In selected patients with CKD, particularly those with advanced kidney disease or concomitant heart failure, fluid overload may also increase body weight and BMI without necessarily reflecting greater adiposity. However, body composition, sarcopenia, and objective volume status were not directly assessed in the present study; therefore, the contribution of these potential mechanisms to the observed association between higher BMI and non-robust status cannot be determined. The physical activity component was assessed using a study-specific three-item approach rather than the original energy expenditure-based Fried criterion and was not formally validated against the original criterion. In a sensitivity analysis excluding this component, the proportion classified as non-robust decreased from 72.4% to 67.5%, although the distribution across frailty categories changed more substantially, with frailty decreasing from 26.4% to 11.0% (Supplementary Table S5). Thus, while the overall predominance of non-robust status was maintained, the absolute frailty prevalence was sensitive to the operational assessment of physical activity. This finding suggests that the study-specific physical activity criterion may have influenced the estimated prevalence of frailty and may limit direct comparison with studies applying the original Fried energy expenditure-based criterion or other definitions of low physical activity.

### 4.6. Frailty in the Context of SGLT-2 Inhibitor Therapy

SGLT-2 inhibitors have become a cornerstone of CKD management because of their well-established kidney and cardiovascular protective effects, which extend beyond glucose lowering and are also observed in patients without diabetes [13,14]. Current international guidelines therefore recommend SGLT-2 inhibitors for eligible patients with CKD to reduce the risk of kidney disease progression and cardiovascular events [13]. Frailty has also been evaluated in analyses of major SGLT-2 inhibitor trials using a cumulative deficit-based frailty index rather than the Fried Physical Frailty Phenotype. In DAPA-HF, which included patients with heart failure with reduced ejection fraction, 15.7% of participants were classified in the highest frailty category, whereas the corresponding proportion was 23.8% in DELIVER, which enrolled patients with heart failure with mildly reduced or preserved ejection fraction [17,18]. These estimates are not directly comparable with the 26.4% frailty prevalence observed in our cohort because of substantial differences in the study populations and, importantly, the method used to define frailty.

In the present study, SGLT-2 inhibitor use defined the clinical population under investigation rather than an exposure whose effect on frailty was being tested. Accordingly, the high prevalence of frailty and pre-frailty should be interpreted as a characterization of physical vulnerability among older adults receiving SGLT-2 inhibitors, rather than as evidence of a treatment effect. In this context, frailty assessment may provide complementary information regarding physical reserve and vulnerability but should not be interpreted as a criterion for initiating or discontinuing SGLT-2 inhibitor therapy.

In our cohort, the distribution of SGLT-2 inhibitor treatment duration did not differ across frailty categories, with 57.8%, 58.7%, and 58.1% of robust, pre-frail, and frail participants, respectively, receiving treatment for >12 months. Given the cross-sectional design, absence of pre-treatment frailty assessment, and lack of a non-SGLT-2 comparator, this descriptive finding cannot establish whether treatment duration is related to frailty.

Because SGLT-2 inhibitors may be associated with modest weight loss, the unintentional weight-loss component of the Fried phenotype warrants particular consideration in this population. In an additional analysis, the frequency of unintentional weight loss did not differ significantly across SGLT-2 inhibitor treatment-duration categories (1–3 months: 6.3%; 3–12 months: 16.7%; >12 months: 14.7%; *p* = 0.40). However, because the timing and cause of weight loss relative to SGLT-2 inhibitor initiation were not systematically recorded, drug-related weight loss could not be reliably distinguished from weight loss attributable to other causes.

Prospective longitudinal studies incorporating baseline and follow-up frailty assessments and an appropriate comparator group are needed to clarify the longitudinal relationship between SGLT-2 inhibitor therapy, treatment duration, and frailty trajectories in older adults with CKD.

### 4.7. Clinical Implications

From a clinical perspective, the high prevalence of frailty and pre-frailty observed in this CKD-predominant cohort highlights the potential value of frailty assessment as part of the broader clinical evaluation of older adults. The observed association between higher BMI and non-robust status further suggests that BMI should not be interpreted in isolation when assessing frailty-related vulnerability, as BMI does not distinguish adiposity from lean mass or directly reflect physical function. A brief phenotype-based assessment of physical vulnerability may provide complementary information during outpatient evaluation, particularly in patients with a high comorbidity burden, anemia, or evidence of functional decline. When frailty or pre-frailty is identified, clinicians may consider a more comprehensive evaluation of potentially contributing and modifiable factors, including physical function, nutritional status, comorbidity burden, anemia, and medication burden. Such an evaluation may inform individualized multidisciplinary assessment and management when clinically appropriate. However, these clinical implications are hypothesis-generating and should be interpreted cautiously given the single-center cross-sectional design, as the present study did not evaluate whether routine frailty screening or frailty-guided interventions improve clinical outcomes.

### 4.8. Strengths and Limitations

This study has several limitations. First, its cross-sectional design precludes causal inference regarding the relationships between frailty and the evaluated clinical variables. Second, the relatively small sample size and single-center design may have limited the statistical power to detect weaker associations and may limit the generalizability of the findings. In addition, recruitment predominantly from a nephrology outpatient setting resulted in a CKD-predominant population with a substantial comorbidity burden, introducing potential selection bias and limiting the generalizability of the observed frailty prevalence to broader populations of older adults receiving SGLT-2 inhibitors. Because detailed baseline characteristics were not available for all screened individuals who were not included in the final cohort, the direction and magnitude of this potential selection bias could not be quantified. Third, the absence of a comparator group of older adults with CKD not receiving SGLT-2 inhibitors precluded direct evaluation of the relationship between SGLT-2 inhibitor therapy and frailty. Accordingly, no treatment-specific conclusions regarding frailty can be drawn from the present study. Frailty status before treatment initiation was also unavailable, limiting interpretation of the relationship between treatment duration and frailty status. Furthermore, low physical activity was assessed using a study-specific, self-reported approach rather than the original energy expenditure-based criterion of the Fried Frailty Phenotype. Although simplified physical activity assessments have been used in previous modified Fried phenotypes, the specific three-item classification and ≥2-of-3 threshold in the present study were prespecified and have not been independently validated against the original Fried physical activity criterion. Sensitivity analysis indicated that frailty classification, particularly the absolute prevalence of frailty, was sensitive to the operational assessment of physical activity; therefore, this methodological difference should be considered when comparing our findings with studies applying the original Fried phenotype. Additional potential sources of selection bias should also be acknowledged. Thirty individuals with previous SGLT-2 inhibitor exposure who were no longer receiving treatment at the time of study assessment were excluded, and the reasons for treatment discontinuation were not systematically recorded. Therefore, the study population represents individuals actively receiving SGLT-2 inhibitor therapy at the time of assessment, and the potential influence of excluding previous users on the observed frailty prevalence cannot be determined. In addition, Sixteen individuals with severe cognitive impairment were excluded because reliable self-report and standardized frailty assessment could not be completed without proxy assessment. This exclusion may have resulted in underrepresentation of more vulnerable older adults and may therefore have influenced the observed prevalence of frailty. The classification of weakness was also sensitive to the grip-strength thresholds applied, which should be considered when interpreting the prevalence estimates and comparing the findings with other populations. Finally, the timing and cause of unintentional weight loss relative to treatment initiation were not systematically recorded; therefore, weight loss potentially related to SGLT-2 inhibitor therapy could not be reliably distinguished from weight loss attributable to other causes.

Despite these limitations, the study has several strengths. Frailty was prospectively assessed using a prespecified, modified Fried Physical Frailty Phenotype, incorporating objective measurements of handgrip strength and walking speed. All anthropometric and physical performance measurements, including body weight, height, body mass index, systolic and diastolic blood pressure, walking speed, and handgrip strength, were performed by a single nephrologist according to a standardized protocol, thereby minimizing inter-observer variability and improving measurement consistency. In addition to categorizing participants as robust, pre-frail, or frail, the distribution of individual frailty components across these categories was examined, allowing a more detailed characterization of the Physical Frailty Phenotype. Sensitivity analyses using alternative operational definitions for key frailty components allowed the influence of different classification approaches on the primary findings to be examined. Furthermore, the inclusion of a well-characterized cohort of older adults actively receiving SGLT-2 inhibitors in a CKD-predominant population provides real-world descriptive data on frailty and its physical components from routine outpatient practice.

## 5. Conclusions

Frailty and pre-frailty were common among older adults receiving SGLT-2 inhibitors in this CKD-predominant population, with only 27.6% of participants classified as robust. Higher BMI and greater comorbidity burden were independently associated with higher odds of non-robust status, whereas higher hemoglobin levels were associated with lower odds. These findings highlight the substantial burden of physical vulnerability in this selected clinical population and support the potential value of frailty assessment as a complementary component of the broader clinical evaluation of older adults, rather than as a tool for determining eligibility for SGLT-2 inhibitor therapy. Given the study design and absence of a comparator group, no treatment-specific conclusions regarding SGLT-2 inhibitor therapy and frailty can be drawn. Prospective longitudinal studies with larger cohorts and appropriate comparator groups are needed to characterize frailty trajectories in older adults with CKD.

## Figures and Tables

**Table 1:** Study inclusion and exclusion criteria.

Inclusion Criteria	Exclusion Criteria
All of the following:	Any of the following:
Age ≥ 60 years	Discontinuation of SGLT-2 inhibitor therapy before study assessment
Receiving an SGLT-2 inhibitor (empagliflozin or dapagliflozin) for ≥1 month	Acute kidney injury and/or active infection at the time of assessment
Provided written informed consent	Severe cognitive impairment precluding reliable self-report during frailty assessment
	Declined participation because of time constraints related to completion of the frailty assessment

**Table 2:** Demographic, clinical and laboratory characteristics of the overall study cohort.

Demographic and Clinical Parameters	
**Age (years), median (Q1–Q3)**	69 (65–73)
**Gender**	***n* (%)**
Male	85 (52.1%)
Female	78 (47.9%)
**Smoking Status**	***n* (%)**
Never	69 (42.3%)
Former smoker	74 (45.4%)
Current smoker	20 (12.3%)
**BMI (kg/m^2^)**	31.44 ± 5.52
**SBP (mmHg)**	130 (110–150)
**DBP (mmHg)**	70 (60–80)
**CCI, median (Q1–Q3)**	3 (2–4)
**Number of medications, mean ± SD**	7.83 ± 2.47
**Comorbidity**	***n* (%)**
DM	143 (87.7%)
HT	143 (87.7%)
CAD	75 (46%)
HF	49 (30.1%)
Atrial Fibrillation	8 (4.9%)
COPD	20 (12.3%)
**Renal and Biochemical Parameters**	**Median (Q1–Q3)**
Creatinine, mg/dL	1.35 (1.06–1.66)
eGFR, mL/min/1.73 m^2^	44 (35.3–62.6)
PCR, mg/g	215 (120–576.5)
ACR, mg/g	57.22 (12–290.6)
Hemoglobin, g/dL	13.3 (11.9–14.9)
Serum albumin, g/L	44.25 (42.38–45.9)
CRP, mg/L	2.97 (1.3–6.6)
NLR	2.2 (1.5–2.7)
**CKD status**	**n/N (%)**
CKD	137/162 (84.6%)
**GFR category**	***n* (%)**
G1	13 (8%)
G2	31 (19%)
G3a	32 (19.6%)
G3b	67 (41.1%)
G4	19 (11.7%)
G5	1 (0.6%)
**SGLT-2 Inhibitors and Concomitant Medications**	***n* (%)**
**Type of SGLT-2 inhibitor**	
Dapagliflozin	72 (44.2%)
Empagliflozin	91 (55.8%)
**Duration of SGLT-2 inhibitor therapy**	***n* (%)**
1–3 months	32 (19.6%)
3–12 months	36 (22.1%)
>12 months	95 (58.3%)
**Concomitant medications, *n* (%)**	
RAS inhibitor	133 (81.6%)
Diuretics	104 (63.8%)
Finerenone	5 (3.1%)

BMI, body mass index; SBP, systolic blood pressure; DBP, diastolic blood pressure; CCI, Charlson Comorbidity Index; DM, diabetes mellitus; HT, hypertension; CAD, coronary artery disease; HF, heart failure; COPD, chronic obstructive pulmonary disease; eGFR, estimated glomerular filtration rate; PCR, urine protein-to-creatinine ratio; ACR, urine albumin-to-creatinine ratio; CRP, C-reactive protein; NLR, neutrophil-to-lymphocyte ratio; CKD, chronic kidney disease; SGLT-2, sodium–glucose cotransporter-2; RAS, renin–angiotensin system. Data are presented using available cases. Denominators were 159 for ACR, 160 for CRP, and 162 for PCR and serum albumin; CKD status was available for 162 participants.

**Table 3:** Distribution of demographic, clinical, and laboratory characteristics according to frailty status.

Demographic and Clinical Parameters	Robust (*n* = 45)	Pre-Frail (*n* = 75)	Frail (*n* = 43)	Effect Size	*p*
**Age (years), median (Q1–Q3)**	69 (65–71)	67 (64–71)	74 (70–77)	ε^2^ = 0.142	<0.001 ^a^
**Gender, *n* (%)**					
Male	28 (62.2)	38 (50.7)	19 (44.2)	V = 0.135	0.224 ^b^
Female	17 (37.8)	37 (49.3)	24 (55.8)		
**BMI, kg/m^2^ (mean ± SD)**	29.68 ± 4.75	32.51 ± 5.22	31.4 ± 6.35	η^2^ = 0.046	0.024 ^c^
**Smoking Status, *n* (%)**					
Never	19 (42.2)	29 (38.7)	21 (48.8)	V = 0.081	0.714 ^b^
Former smoker	19 (42.2)	38 (50.7)	17 (39.5)		
Current smoker	7 (15.6)	8 (10.7)	5 (11.6)		
**SBP, mmHg, median (Q1–Q3)**	130 (115–150)	135 (120–150)	120 (110–140)	ε^2^ = 0.039	0.043 ^a^
**DBP, mmHg, median (Q1–Q3)**	70 (60–82.5)	70 (60–80)	70 (50–80)	ε^2^ = 0.009	0.485
**CCI, median (Q1–Q3)**	2 (1–3)	3 (2–4)	4 (3–6)	ε^2^ = 0.139	<0.001 ^a^
**Number of medications**	7.13 ± 2.41	7.97 ± 2.49	8.33 ± 2.38	η^2^ = 0.035	0.060 ^c^
**Comorbidity**	**N (%)**				
DM	37 (82.2%)	66 (88%)	40 (93%)	V = 0.121	0.302 ^b^
HT	36 (80%)	66 (88%)	41 (95.3%)	V = 0.172	0.090 ^b^
CAD	18 (40%)	33 (44%)	24 (55.8%)	V = 0.122	0.295 ^b^
HF	9 (20%)	20 (26.7%)	20 (46.5%)	V = 0.223	0.017 ^b^
Atrial Fibrillation	2 (4.4%)	2 (2.7%)	4 (9.3%)	V = 0.126	0.260 ^d^
COPD	3 (6.7%)	7 (9.3%)	10 (23.3%)	V = 0.203	0.034 ^b^
**Renal and Biochemical Parameters**		median (Q1–Q3)			
Creatinine, mg/dL	1.31 (1.08–1.57)	1.31(0.99–1.66)	1.59(1.07–1.76)	ε^2^ = 0.020	0.205 ^a^
eGFR, mL/min/1.73 m^2^	49.4 (39.75–62.7)	47.5 (36–63.1)	40.5 (30.1–57.8)	ε^2^ = 0.036	0.056 ^a^
PCR, mg/g	180 (120–330)	160 (117.5–525)	400 (180–850)	ε^2^ = 0.072	0.003 ^a^
ACR, mg/g	30.09 (10.98–112.91)	26.29 (10–296.29)	122.56 (48.91–485.23)	ε^2^ = 0.053	0.014 ^a^
Hemoglobin, g/dL	14.3 (13–15.6)	13.1 (12–14.5)	12.1 (10.6–14.8)	ε^2^ = 0.094	<0.001 ^a^
Serum albumin, g/L	45.3 (44.15–46.75)	44.2 (42.75–46.4)	43.6 (40.3–44.8)	ε^2^ = 0.122	<0.001 ^a^
CRP, mg/L	1.9 (1.05–4.97)	3.3 (1.38–7.73)	2.3 (1.38–6.77)	ε^2^ = 0.019	0.220 ^a^
NLR	2.08 (1.44–2.7)	2.04 (1.46–2.63)	2.48 (2.03–3.21)	ε^2^ = 0.054	0.013 ^a^
**SGLT-2 Inhibitors and Concomitant Medications**					
**Type of SGLT-2 inhibitor**					
Dapagliflozin	20 (44.4%)	37 (49.3%)	15 (34.9%)	V = 0.119	0.314 ^b^
Empagliflozin	25 (55.6%)	38 (50.7%)	28 (65.1%)		
**Duration of SGLT-2 inhibitor therapy, *n* (%)**					
1–3 months	9 (20%)	15 (20%)	8 (18.6%)	V = 0.016	0.999 ^b^
3–12 months	10 (22.2%)	16 (21.3%)	10 (23.3%)		
>12 months	26 (57.8%)	44 (58.7%)	25 (58.1%)		
**Concomitant medications, *n* (%)**					
RAS inhibitor	37 (82.2%)	63 (84%)	33 (76.7%)	V = 0.077	0.614 ^b^
Diuretics	27 (60%)	46 (61.3%)	31 (72.1%)	V = 0.104	0.415 ^b^
Finerenone	1 (2.2%)	4 (5.3%)	0	V = 0.130	0.383 ^d^
**Albuminuria category, *n* (%)**					
**A1 (<30 mg/g)**	22 (50%)	38 (52.1%)	9 (21.4%)	V = 0.202	0.011 ^b^
**A2 (30–300 mg/g)**	13 (29.5%)	17 (23.3%)	21 (50%)		
**A3 (>300 mg/g)**	9 (20.5%)	18 (24.7%)	12 (28.6%)		

BMI, body mass index; SBP, systolic blood pressure; DBP, diastolic blood pressure; CCI, Charlson Comorbidity Index; DM, diabetes mellitus; HT, hypertension; CAD, coronary artery disease; HF, heart failure; COPD, chronic obstructive pulmonary disease; eGFR, estimated glomerular filtration rate; PCR, urine protein-to-creatinine ratio; ACR, urine albumin-to-creatinine ratio; CRP, C-reactive protein; NLR, neutrophil-to-lymphocyte ratio; SGLT-2, sodium–glucose cotransporter-2; RAS, renin–angiotensin system. ^a^, Kruskal–Wallis test; ^b^, Pearson chi-square test; ^c^, ANOVA; ^d^, Fisher–Freeman–Halton test. Effect sizes: η^2^, eta squared (ANOVA); ε^2^, epsilon squared (Kruskal–Wallis test); V, Cramér’s V (categorical variables). Thresholds: η^2^/ε^2^, 0.01 small, 0.06 medium, and 0.14 large; Cramér’s V, 0.10 small, 0.30 medium, and 0.50 large. Analyses were performed using available cases; denominators may therefore vary slightly across variables because of missing data, as detailed in Supplementary Table S3.

**Table 4:** Comparison of demographic, clinical, and laboratory characteristics between robust and non-robust participants.

	Frailty Status			
Demographic, Clinical, and Laboratory Parameters	Robust (*n* = 45)	Non-Robust (*n* = 118)	Effect Size	*p* Value
**Age (years), median (Q1–Q3)**	69 (65–71)	69.5 (64–74)	r = 0.066	0.397 ^e^
**CCI, median (Q1–Q3)**	2 (1–3)	3 (3–5)	r = 0.258	0.001 ^e^
**HF, *n* (%)**	9 (20%)	40 (33.9%)	φ = 0.136	0.084 ^b^
**COPD, *n* (%)**	3 (6.7%)	17 (14.4%)	φ = 0.105	0.178 ^b^
**PCR, mg/g**	180 (120–330)	230 (130–647.5)	r = 0.089	0.257 ^e^
**ACR, mg/g**	30.09 (10.98–112.91)	67.93 (12–353)	r = 0.090	0.253 ^e^
**Hemoglobin, g/dL, mean ± SD**	14.23 ± 1.74	12.96 ± 1.99	d = 0.66	<0.001 ^f^
**Serum albumin, g/L**	45.3 (44.15–46.75)	43.9 (41.5–45.65)	r = 0.258	0.001 ^e^
**NLR, median (Q1–Q3)**	2.08 (1.44–2.7)	2.24 (1.6–2.87)	r = 0.098	0.212 ^e^
**Renal Parameters**				
**eGFR, mL/min/1.73 m^2^, median (Q1–Q3)**	49.4 (36.75–62.7)	43.65 (34.28–61.68)	r = 0.089	0.257 ^e^
**GFR category**				
G1	3 (6.7%)	10 (8.5%)	V = 0.155	0.593 ^d^
G2	10 (22.2%)	21 (17.8%)		
G3a	12 (26.7%)	20 (16.9%)		
G3b	17 (37.8%)	50 (42.4%)		
G4	3 (6.7%)	16 (13.6%)		
G5	0	1 (0.8%)		
**CKD status, *n* (%)**	37 (82.2%)	100 (85.5%)	φ = 0.040	0.608 ^b^
**Albuminuria category, *n* (%)**				
**A1 (<30 mg/g)**	22 (50%)	47 (40.9%)	V = 0.085	0.564 ^b^
**A2 (30–300 mg/g)**	13 (29.5%)	38 (33%)		
**A3 (>300 mg/g)**	9 (20.5%)	30 (26.1%)		
**SGLT-2 inhibitor**				
Dapagliflozin	20 (44.4%)	52 (44.1%)	φ = 0.003	0.965 ^b^
Empagliflozin	25 (55.6%)	66 (55.9%)		
**Duration of SGLT-2 inhibitor therapy**				
1–3 months	9 (20%)	23 (19.5%)	V = 0.007	0.996 ^b^
3–12 months	10 (22.2%)	26 (22%)		
>12 months	26 (57.8%)	69 (58.5%)		

Data are presented as mean ± SD, median (Q1–Q3), or *n* (%), as appropriate. SD, standard deviation; CCI, Charlson Comorbidity Index; HF, heart failure; COPD, chronic obstructive pulmonary disease; PCR, urine protein-to-creatinine ratio; eGFR, estimated glomerular filtration rate; CKD, chronic kidney disease; NLR, neutrophil-to-lymphocyte ratio; SGLT-2, sodium–glucose cotransporter-2. ^b^, Pearson chi-square test; ^d^, Fisher–Freeman–Halton test; ^e^, Mann–Whitney U test; ^f^, independent two-sample *t*-test. Effect sizes: r = |Z|/√N (Mann–Whitney U test); d, Cohen’s d (independent *t*-test); φ, phi coefficient (2 × 2 tables); V, Cramér’s V (larger contingency tables). Thresholds: r and φ/V, 0.10 small, 0.30 medium, and 0.50 large; d, 0.20 small, 0.50 medium, and 0.80 large. Analyses were performed using available cases; denominators may therefore vary slightly across variables because of missing data, as detailed in Supplementary Table S3.

**Table 5:** Univariate and multivariate logistic regression analyses of factors associated with non-robust status.

	Univariate Model		Multivariate Model	
	OR (95% CI)	*p*	aOR (95% CI)	*p*
Age (years)	1.02 (0.96–1.08)	0.495	1.01 (0.95–1.07)	0.781
Sex (male)	0.57 (0.28–1.15)	0.114	1.28 (0.51–3.21)	0.600
BMI (per kg/m^2^)	1.09 (1.02–1.18)	0.014	1.12 (1.03–1.22)	0.010
CCI	1.47 (1.16–1.85)	0.001	1.42 (1.12–1.81)	0.004
Hemoglobin (per g/dL)	0.70 (0.58–0.86)	<0.001	0.72 (0.57–0.91)	0.007

Dependent variable: non-robust status (pre-frail and frail participants combined, *n* = 118) versus robust status (reference, *n* = 45). Age and sex were forced into the multivariable model; CCI was entered instead of individual comorbidities to avoid collinearity. Full univariate results are presented in Supplementary Table S2. Model statistics: omnibus χ^2^ = 30.16, df = 5, *p* < 0.001; Nagelkerke R^2^ = 0.244; Hosmer–Lemeshow χ^2^ = 5.58, df = 8, *p* = 0.695; overall correct classification = 77.9%; AUC = 0.761 (95% CI 0.681–0.842). OR, odds ratio; aOR, adjusted odds ratio; CI, confidence interval; BMI, body mass index; CCI, Charlson Comorbidity Index; AUC, area under the receiver operating characteristic curve.

## Data Availability

The data presented in this study are available from the corresponding author upon reasonable request.

## Supplementary Materials

The following supporting information can be downloaded at: https://www.mdpi.com/article/10.3390/medicina62101938/s1, Table S1: Study-Specific Assessment and Scoring Algorithm for the Low Physical Activity Component; Table S2: Univariate logistic regression analysis of factors associated with non-robust status; Table S3: Completeness of data for key study variables; Table S4: Distribution of weakness according to sex- and BMI-specific strata of the Fried phenotype; Table S5: Sensitivity analyses of frailty classification using alternative operational definitions; Table S6: Sensitivity analysis of factors associated with non-robust status after additional adjustment for albuminuria; Table S7: Sensitivity analyses of factors associated with frailty status.

## Abbreviations

The following abbreviations are used in this manuscript:

ACR	urine albumin-to-creatinine ratio
BMI	body mass index
CAD	coronary artery disease
CCI	Charlson Comorbidity Index
CES-D	Center for Epidemiologic Studies Depression scale
CI	confidence interval
CKD	chronic kidney disease
CKD-EPI	Chronic Kidney Disease Epidemiology Collaboration
COPD	chronic obstructive pulmonary disease
CRP	C-reactive protein
CVD	cardiovascular disease
DBP	diastolic blood pressure
DM	diabetes mellitus
eGFR	estimated glomerular filtration rate
GFR	glomerular filtration rate
HF	heart failure
NLR	neutrophil-to-lymphocyte ratio
OR	odds ratio
PCR	urine protein-to-creatinine ratio
Q1	first quartile
Q3	third quartile
RAS	renin–angiotensin system
SBP	systolic blood pressure
SD	standard deviation
SGLT-2	sodium–glucose cotransporter-2
T2DM	type 2 diabetes mellitus
