# Supplementary material for: Frailty Phenotype Among Older Adults Receiving SGLT-2 Inhibitors in a CKD-Predominant Population: A Prospective Cross-Sectional Study
Source: Medicina (Kaunas). 2026 Oct 8;62(10):1938. doi: 10.3390/medicina62101938 (PMC13707250; doi:10.3390/medicina62101938)
Supplement: Supplementary file 1 [file medicina-62-01938-s001.zip › medicina-4570704-supplementary.pdf]

Supplementary Table S1. Study-Specific Assessment and Scoring Algorithm for the Low Physical Activity Component

Item	Physical Activity Question	Response Indicating Low Physical Activity	Score
1	Had you walked for at least 20–30 minutes on ≥5 days during the preceding week?	No	1
2	Were you generally active in daily activities such as housework, shopping, and going outdoors?	No	1
3	Did you spend most of the day sitting or lying down?	Yes	1

Scoring algorithm: Each indicator of low physical activity was assigned 1 point. Participants meeting at least two of the three indicators (≥2/3) were classified as having low physical activity. This study-specific scoring algorithm was prespecified in the study protocol and has not been validated against the original energy expenditure-based physical activity criterion of the Fried phenotype.

Supplementary Table S2. Univariate logistic regression analysis of factors associated with non-robust status

Variable	OR (95% CI)	p value
Age (per year)	1.02 (0.96–1.08)	0.495
Sex (male vs female)	0.57 (0.28–1.15)	0.114*
BMI (per kg/m²)	1.09 (1.02–1.18)	0.014*
Hypertension	2.43 (0.93–6.34)	0.069*
Diabetes mellitus	1.91 (0.72–5.04)	0.191*
Heart failure	2.05 (0.90–4.68)	0.087*
COPD/asthma	2.36 (0.66–8.47)	0.189*
CCI (per point)	1.47 (1.16–1.85)	0.001*
Number of medications	1.18 (1.02–1.38)	0.027*
Hemoglobin (per g/dL)	0.70 (0.58–0.86)	<0.001*
Serum albumin (per g/L)	0.82 (0.72–0.93)	0.002*
NLR	1.21 (0.86–1.70)	0.276
eGFR (per mL/min/1.73 m²)	0.99 (0.98–1.01)	0.443
log-ACR	1.13 (0.93–1.37)	0.217*

Variable	OR (95% CI)	p value
log-PCR	1.27 (0.90–1.79)	0.169*

Dependent variable: non-robust status (pre-frail and frail participants combined, n=118) versus robust status (reference, n=45).

*Variables with p<0.25 in univariate analysis were considered candidates for the multivariable model.

BMI: body mass index; CCI: Charlson Comorbidity Index; COPD: chronic obstructive pulmonary disease; eGFR: estimated glomerular filtration rate; ACR: urine albumin-to-creatinine ratio; PCR: urine protein-to-creatinine ratio; OR: odds ratio; CI: confidence interval.

Supplementary Table S3. Completeness of data for key study variables

Variable	Available (n)	Missing (n)	Completenes s (%)
Age, sex, BMI, and blood pressure	163	0	100
Comorbidities (DM, HT, CAD, HF, COPD, and AF)	163	0	100
Smoking status and number of medications	163	0	100
Frailty phenotype components	163	0	100
Hemoglobin and NLR	163	0	100
Serum creatinine and eGFR	163	0	100
Spot urine PCR	162	1	99.4
Serum albumin	162	1	99.4
CRP	160	3	98.2
Spot urine ACR	159	4	97.5

Abbreviations: BMI, body mass index; DM, diabetes mellitus; HT, hypertension; CAD, coronary artery disease; HF, heart failure; COPD, chronic obstructive pulmonary disease; AF, atrial fibrillation; NLR, neutrophil-to-lymphocyte ratio; eGFR, estimated glomerular filtration rate; PCR, protein-to-creatinine ratio; CRP, C-reactive protein; ACR, albumin-to-creatinine ratio.

Supplementary Table S4. Distribution of weakness according to sex- and BMI-specific strata of the Fried phenotype

Sex	BMI stratum (kg/m²)	Total, N	Weakness, n (%)
Men	≤24	10	6 (60.0)
Men	24.1–26.0	4	1 (25.0)
Men	26.1–28.0	19	5 (26.3)
Men	>28.0	52	31 (59.6)
Women	≤23	0	0 (—)
Women	23.1–26.0	6	4 (66.7)
Women	26.1–29.0	15	5 (33.3)
Women	>29.0	57	34 (59.6)
**Total**	**—**	**163**	**86 (52.8)**

Weakness values reflect the classification used in the primary Fried phenotype analysis. BMI, body mass index.

Supplementary Table S5. Sensitivity analyses of frailty classification using alternative operational definitions. (n=163)

Frailty status	Primary modified Fried phenotype, n (%) [95% CI]	Excluding the physical activity component, n (%) [95% CI]	Using Turkish handgrip strength cut-offs, n (%) [95% CI]
Robust	45 (27.6; 21.3–34.9)	53 (32.5; 25.8–40.0)	62 (38.0; 30.9–45.7)
Pre-frail	75 (46.0; 38.5–53.7)	92 (56.4; 48.8–63.8)	71 (43.6; 36.2–51.2)
Frail	43 (26.4; 20.2–33.6)	18 (11.0; 7.1–16.8)	30 (18.4; 13.2–25.1)
Non-robust (pre-frail + frail)	118 (72.4; 65.1–78.7)	110 (67.5; 60.0–74.2)	101 (62.0; 54.3–69.1)

Values are presented as n (%; 95% CI). Confidence intervals were calculated using the Wilson method.

Primary modified Fried phenotype: five components, with weakness defined using sex- and BMI-stratified cut-offs; robust, 0 components; pre-frail, 1–2 components; frail, ≥3 components. Excluding physical activity: frailty status was reclassified using the remaining four components (unintentional weight loss, exhaustion, weakness and slow gait speed); frail, ≥3 of four components. Turkish grip strength cut-offs: weakness redefined as grip strength ≤27.7 kg in men and ≤13.6 kg in women, with all five components retained; weakness prevalence decreased from 52.8% to 23.3%.

Among the 75 pre-frail participants under the **primary modified Fried phenotype,** 28 (37.3%) met only the weakness criterion, corresponding to 23.7% of non-robust participants.

CI: confidence interval.

Supplementary Table S6. Sensitivity analysis of factors associated with non-robust status after additional adjustment for albuminuria

Variable	Main model (n=163)		Sensitivity model (n=159)	
	aOR (95% CI)	p	aOR (95% CI)	p
Age (per year)	1.01 (0.95–1.07)	0.781	1.03 (0.96–1.10)	0.426
Sex (male vs female)	1.28 (0.51–3.21)	0.600	1.11 (0.43–2.85)	0.834
BMI (per kg/m²)	1.12 (1.03–1.22)	**0.010**	1.12 (1.02–1.22)	**0.013**
CCI (per point)	1.42 (1.12–1.81)	**0.004**	1.42 (1.11–1.82)	**0.005**
Hemoglobin (per g/dL)	0.72 (0.57–0.91)	**0.007**	0.75 (0.59–0.96)	**0.021**

Variable	Main model (n=163)		Sensitivity model (n=159)	
	aOR (95% CI)	p	aOR (95% CI)	p
log-ACR	—	—	1.07 (0.86–1.33)	0.534

Dependent variable: non-robust status (pre-frail and frail participants combined) versus robust status (reference). The sensitivity model additionally included log-ACR; four participants with missing ACR data were excluded from this model. Bold values indicate $p<0.05$. "—" indicates that log-ACR was not included in the main model; its univariate association is presented in Supplementary Table S5.

Main model: Hosmer–Lemeshow p=0.695; Nagelkerke R^2=0.244; AUC 0.761 (95% CI 0.681–0.842). Sensitivity model: Hosmer–Lemeshow p=0.396; Nagelkerke R^2=0.241.

aOR: adjusted odds ratio; CI: confidence interval; BMI: body mass index; CCI: Charlson Comorbidity Index; ACR: urine albumin-to-creatinine ratio; AUC: area under the receiver operating characteristic curve.

Supplementary Table S7. Sensitivity analyses of factors associated with frailty status

Variable	Main model (n=163)	Excluding indeterminate CKD status (n=162)	Participants meeting CKD criteria (n=137)	Excluding observations with Cook's distance >4/n (n=150)	Ordinal model, Fried score 0–5 (n=163)
Age (per year)	1.01 (0.95–1.07)<br>p = 0.781	1.01 (0.95–1.07)<br>p = 0.754	1.01 (0.95–1.08)<br>p = 0.725	1.06 (0.97–1.15)<br>p = 0.207	1.06 (1.01–1.12)<br>**p = 0.015**
Sex (male vs female)	1.28 (0.51–3.21)<br>p = 0.600	1.29 (0.52–3.25)<br>p = 0.584	1.02 (0.37–2.77)<br>p = 0.971	1.43 (0.44–4.62)<br>p = 0.547	0.98 (0.49–1.99)<br>p = 0.962
BMI (per kg/m^2)	1.12 (1.03–1.22)<br>**p = 0.010**	1.12 (1.03–1.22)<br>**p = 0.009**	1.11 (1.01–1.22)<br>**p = 0.034**	1.20 (1.07–1.35)<br>**p = 0.002**	1.04 (0.98–1.10)<br>p = 0.174
CCI (per point)	1.42 (1.12–1.81)<br>**p = 0.004**	1.41 (1.11–1.80)<br>**p = 0.005**	1.47 (1.13–1.93)<br>**p = 0.005**	1.83 (1.30–2.58)<br>**p = 0.001**	1.41 (1.20–1.66)<br>**p <0.001**
Hemoglobin (per g/dL)	0.72 (0.57–0.91)<br>**p = 0.007**	0.72 (0.57–0.92)<br>**p = 0.007**	0.74 (0.57–0.95)<br>**p = 0.020**	0.60 (0.43–0.84)<br>**p = 0.002**	0.78 (0.65–0.94)<br>**p = 0.008**

Values are adjusted odds ratios (95% CI) and p values. In the first four models, the dependent variable was non-robust status (pre-frail and frail combined) versus robust status (reference). In the ordinal model, the dependent variable was the Fried frailty score (0–5), analysed using ordinal logistic regression; odds ratios represent the odds of a higher frailty score. All models included age, sex, BMI, CCI and hemoglobin. Bold p values indicate $p<0.05$.

No covariate data were missing in any model. Influential observations were assessed using Cook's distance, leverage and studentized residuals; the maximum Cook's distance in the main model was 0.05.

CI: confidence interval; BMI: body mass index; CCI: Charlson Comorbidity Index; CKD: chronic kidney disease.
